# The social underpinnings of mental distress in the time of COVID-19 – time for urgent action

**DOI:** 10.12688/wellcomeopenres.16123.1

**Published:** 2020-07-13

**Authors:** Nikolas Rose, Nick Manning, Richard Bentall, Kamaldeep Bhui, Rochelle Burgess, Sarah Carr, Flora Cornish, Delan Devakumar, Jennifer B. Dowd, Stefan Ecks, Alison Faulkner, Alex Ruck Keene, James Kirkbride, Martin Knapp, Anne M. Lovell, Paul Martin, Joanna Moncrieff, Hester Parr, Martyn Pickersgill, Genevra Richardson, Sally Sheard

**Affiliations:** 1 Centre for Society and Mental Health, King's College London, London, WC2B 4BG, UK; 2 Department of Psychology,, University of Sheffield, Sheffield, S1 2LT, UK; 3 Department of Psychiatry, University of Oxford, Oxford, OX3 7JX, UK; 4 Institute for Global Health, University College, London, WC1N 1EH, UK; 5 Institute for Mental Health, University of Birmingham, UK, Birmingham, B15 2TT, UK; 6 Methodology Institute, London School of Economics and Political Science, London, WC2A 2AE, UK; 7 Leverhulme Centre for Demographic Science, University of Oxford, Oxford, OX1 1JD, UK; 8 Department of Anthropology, University of Edinburgh, Edinburgh, EH8 9LD, UK; 9 Survivor Researcher and Trainer, Independent Researcher, London, UK; 10 39 Essex Chambers, 81 Chancery Lane, London, WC2A 1DD, UK; 11 Department of Psychiatry, University College, London, WC1N 1EH, UK; 12 Care Policy and Evaluation Centre, London School of Economics and Political Science, London, WC2A 2AE, UK; 13 CERMES3, Ecole des Hautes Etudes en Sciences Sociales, Paris, 75006, France; 14 Department of Sociology, University of Sheffield, Sheffield, S10 2TN, UK; 15 Department of Geography, University of Glasgow, Glasgow, G12 8QQ, UK; 16 Usher Institute, University of Edinburgh, Edinburgh, EH8 9LD, UK; 17 School of Law, King's College London, London, WC2R 2LS, UK; 18 Department of Public Health, Policy and Systems, University of Liverpool, Liverpool, L69 3GB, UK

**Keywords:** Mental distress, social disadvantage, BAME, universal basic income, benefit system reform

## Abstract

We argue that predictions of a ‘tsunami’ of mental health problems as a consequence of the pandemic of coronavirus disease 2019 (COVID-19) and the lockdown are overstated; feelings of anxiety and sadness are entirely normal reactions to difficult circumstances, not symptoms of poor mental health.  Some people will need specialised mental health support, especially those already leading tough lives; we need immediate reversal of years of underfunding of community mental health services.  However, the disproportionate effects of COVID-19 on the most disadvantaged, especially BAME people placed at risk by their social and economic conditions, were entirely predictable. Mental health is best ensured by urgently rebuilding the social and economic supports stripped away over the last decade. Governments must pump funds into local authorities to rebuild community services, peer support, mutual aid and local community and voluntary sector organisations.  Health care organisations must tackle racism and discrimination to ensure genuine equal access to universal health care.  Government must replace highly conditional benefit systems by something like a universal basic income. All economic and social policies must be subjected to a legally binding mental health audit. This may sound unfeasibly expensive, but the social and economic costs, not to mention the costs in personal and community suffering, though often invisible, are far greater.

## Disclaimer

The views expressed in this article are those of the author(s). Publication in Wellcome Open Research does not imply endorsement by Wellcome.

## Introduction

There has been much discussion about the mental health implications of coronavirus disease 2019 (COVID-19) - both of the pandemic itself and of the ‘lockdown’. Many have predicted short, medium, and long-term mental health problems. There is some belated recognition of the crucial role of social inequality, and the disproportionate toll born by the most disadvantaged groups in society. However, the main emphasis has been on expanding access to specialist mental health services to cope with an anticipated surge in mental health problems. As members of the Society and Mental Health COVID-19 Expert Group, hosted by the Centre for Society and Mental Health at King’s College London, we argue that there is an urgent need for an alternative approach.

Some surveys have reported increased levels of anxiety and sadness and attributed those to the pandemic ^1, 2^. These are normal and understandable responses to situations involving threats and disruptions to habitual forms of life; the curtailing of social contacts and increased social isolation; and encounters - both actual and virtual - with sickness and death. Though undoubtedly distressing, for most people these are not symptoms of mental disorder and will not lead to enduring mental health problems requiring specialist therapeutic intervention. As successful public health interventions during previous crises have shown, the most effective support for those who experience such distress is practical. This includes information to support immediate problem-solving, assistance with everyday tasks, ensuring financial and housing security, maintaining trust by openness and honesty, and, crucially, the (re)building of community infrastructures and informal social support networks ^3^.

But when it comes to mental health, as with so many other dimensions of COVID-19, we are not ‘all in it together’. As so clearly shown by a whole body of evidence on the social determinants of mental health, the greatest risk of developing serious and enduring mental distress will fall upon those already impacted by social inequality, and this will be exacerbated by the current crisis and its aftermath ^4^. Elevated risks of poor psychological wellbeing for the already vulnerable are linked to isolation, economic stress, stigma, racism and social exclusion ^5^ which will be exacerbated as resources are further diverted by COVID-19 responses. Further, we know that physical and mental health are interdependent and entwined, and thus mental health will be affected by the experience of COVID-19 ^6^. There are clear gender implications of COVID-19, and while reports have largely focused on the increased mortality among men, there has been almost no attention to the double burden that the lockdown has imposed on the mental health of women from the most disadvantaged communities many of whom have increased domestic responsibilities while at the same time being obliged to continue paid employment often in front-line jobs. Those experiencing the greatest social disadvantage are thus most likely to suffer the worst mental health impacts, and those with pre-existing mental health conditions may experience a deterioration in their mental health exacerbated by a further reduction in levels of social support available to them.

In our view, such evidence from the social sciences, which is born out by the knowledge of those with lived experience of mental ill health, should have been central to pandemic preparedness planning. We believe that it must now urgently be deployed to identify the places and communities that need most support. Resources must be rapidly, preemptively and unconditionally directed to address immediate material requirements, and strengthen both informal and formal support networks. Interventions such as those proposed by Holmes *et al.* ^7, 8^ based in psychology, psychiatry, pharmacology, genetics, molecular biology, neurology, neuroscience, cognitive sciences, computer science, and mathematics will be ineffective if they do not address the underlying social causes of mental ill health.

Immediate action should be taken to tackle the conditions that impact directly on the most socially excluded, especially Black, Asian, and minority ethnic (BAME) communities. These include poor and overcrowded housing conditions; the experience of racism, xenophobia and violence; obesogenic, degraded and polluted environments; financial insecurity, callous conditional welfare benefits; precarious work, exposed conditions for front line workers in care homes, transport workers, delivery drivers, warehouse packers and taxi drivers; children’s education damaged by schools impoverished by a decade of financial restrictions and lack of access to the resources for digital education, and community facilities hollowed out by a decade of austerity. Hasty policies, such as the curtailing of the rights of mental health patients to proper assessments before involuntary detention as included in the Coronavirus Act 2020, should rapidly be reversed. The social realities impacting mental health will not disappear when lockdown eases. They will only be intensified as the economic consequences of the pandemic play out.

We welcome the publication of the Public Health England review of *Disparities in the Risk and Outcomes of COVID-19*, which shows very clearly the impact of COVID-19 on those most socially disadvantaged ^9^, and note that our argument is supported by the belated publication of the literature reviews and especially the stakeholder input ^10^. The epidemiological evidence confirms that excess burden of COVID-19 born by those from Black and minority ethnic backgrounds is largely accounted for by the dimensions of social disadvantage that we have noted, and this is powerfully reinforced by the contributions of community organizations and mental health service users. If we are to implement policies which bring about progressive and transformative improvements in the mental wellbeing of our most disadvantaged communities as we enter the next phase of recovery from the pandemic, it is critical that the expertise of social scientists, and of those with lived experience of mental ill health, play a key role in policy development and implementation.

This evidence on the social substrates of poor mental health has important lessons for the short, medium, and long-term policies needed to mitigate the transition of understandable distress to significant and enduring mental health problems. Mental health and well-being is enhanced by elevated social solidarity, informal social support, mutual aid and mutual innovation in relation to crisis conditions ^11^, by measures to increase equality ^12^, and by providing the resources necessary for the realization of capabilities ^13,14^. As we set out in Table 1, to create “the optimum structure for mentally healthy life” ^7^ we must harness resources from sociology, anthropology, geography, politics, and economics to inform rapid policy innovation, alongside legal changes, which will, on the one hand, address the fundamental social causes of mental ill health, and, on the other, create the social conditions that maximize human well-being.

**Table 1.** Mental health for all - building back better, building back fairer.

Promoting mental health	Addressing mental illness
Introduce mental health audits and inequality impact assessments of pandemic and post-pandemic policies across all sectors. Replace conditional welfare support with unconditional measures that promote capabilities for the most disadvantaged, such as free, accessible public transport. Ensure sustained adequate support for children from disadvantaged families being ‘home schooled’ including access to meals, breakfast clubs, facilities for internet access and resources for digital education. Design economic policies to maintain a strong safety net of income security, particularly within the most traditionally vulnerable groups, including a - recovery-basic income package which will support all, including the most financially disadvantaged. Ensure equality in access to health services by taking immediate and effective action to tackle institutional racism and to promote anti-racist and inclusive decision-making and practice. Address gender-based discrimination and promoting equal access for lesbian, gay, bisexual and transgender people and people with disabilities. Rapid investment to support mutual aid, community groups and voluntary sector organizations decimated by a decade of austerity, with an emphasis on; women's refuges, homeless charities, community-based support by and for black and minority ethnic people.	Rapid investment in local community facilities and services - local authority 'community and voluntary sector organizations - across a range of health and social sectors. Reverse the rolling back of service users' rights to health and social care services that occurred in pandemic legislation. Re-Invest in community mental health teams, rebuild public mental health infrastructure and community mental health services. Provide resources to support service user and survivor, carer, mutual aid and self-help groups.

 The fault-lines in British society have been starkly disclosed by the pandemic. To ‘build back better’ in the long aftermath of COVID-19, we need to create the social and material environments that not only address the causes of mental ill health but also enhance the capabilities of all citizens to create lives of meaning and purpose for themselves.

## Data availability

### Underlying data

No data is associated with this article.
