# Supplementary material for: The social underpinnings of mental distress in the time of COVID-19 – time for urgent action
Source: Wellcome Open Res. 2020 Jul 13;5:166. [Version 1] doi: 10.12688/wellcomeopenres.16123.1 (PMC7411522; doi:10.12688/wellcomeopenres.16123.1)
Supplement: Peer review history

# Peer review: Reviewer response for version 1

**DOI:** 10.21956/wellcomeopenres.17697.r39807

**Published:** 2020-08-24

**Authors:** Ewen Speed

**Affiliations:** 1 School of Health and Social Care, University of Essex, Colchester, UK

This is a timely and important letter. One of the most important and central features of this letter is the authors insistence on the normality of increased levels of anxiety and sadness and their refusal to regard this as reflective of an underlying ‘tsunami’ of mental health problems. This is entirely correct and this message needs to be applauded and amplified across the range of public health and health policy settings. Similarly I agree entirely with the authors insistence on the important of the impact, overlap and interplay (the intersection) of enduring inequalities and disadvantages in relation to ongoing levels of mental health within the population at large. Furthermore their insistence that these issues must be addressed in terms of questions of ethnicity, gender and labour market participation are also accurate and apposite. However, I think the most important point that they make, and it is one that is frequently overlooked, is the impact that the pandemic conditions will have on people who are already impacted by social inequality. The conditions and consequences of lockdown have clearly exacerbated already existing inequalities, and it is in these instances that, it seems to me, there is the strongest potential for an elevated risk of poor psychological wellbeing. 

 The authors are correct to identify the need for a range of initiatives that enable these marginalised people to improve their situations with a view to the realization of respective capabilities. In this sense, I regard this letter as a very useful and timely provocation, but I feel there is a need to think about what to do next, on foot of this provocation. There is a need to develop ways of thinking about how societies might move towards the realization of respective capabilities post-lockdown. Central to this is the need to find ways to involve these respective groups in developing their own responses to these enduring inequalities and disadvantages (in a spirit of democratic participation and involvement). These responses must be responses that challenge those inequalities and disadvantages at all levels, across everyday experience, across policy and practice, and in terms of ideological critique and more besides. 

 Involvement of those people most directly affected by these inequalities is crucial in beginning to move to address these inequalities, but these inequalities are not in and of themselves new. For example, the inability of precariously employed people to take time off due to sickness is not a new development of the COVID-19 pandemic. However, the public health implications of this situation, in the context of a global infectious disease pandemic are, and this novelty offers an opportunity for progressive change – if not for the protection of individual workers (contemporary capitalism appears, to date, immune to this critique), then for the protection of society as a whole. These are the types of arguments it becomes possible to make, and these are types of arguments that need to be developed. This letter and the thinking that underpins it can play a crucial role in provoking the development of such arguments.

Does the article adequately reference differing views and opinions?

Yes

Are all factual statements correct, and are statements and arguments made adequately supported by citations?

Yes

Is the Open Letter written in accessible language?

Yes

Where applicable, are recommendations and next steps explained clearly for others to follow?

Yes

Is the rationale for the Open Letter provided in sufficient detail?

Yes

Reviewer Expertise:

Medical sociology, health policy, participation and involvement.

I confirm that I have read this submission and believe that I have an appropriate level of expertise to confirm that it is of an acceptable scientific standard.

# Peer review: Reviewer response for version 1

**DOI:** 10.21956/wellcomeopenres.17697.r39764

**Published:** 2020-08-18

**Authors:** Darrin Hodgetts, Clifford Van Ommen, Denise Blake, Veronica Hopner, Mohi Rua, Shiloh Groot, Stuart Carr

**Affiliations:** 1 School of Psychology, Massey University, Wellington, New Zealand; 2 Massey University, Palmerston North, New Zealand; 3 University of Waikato, Hamilton, New Zealand; 4 University of Auckland, Auckland, New Zealand

Rose and colleagues are prominent scholars who have brought insights from across the social and health sciences to promote a compelling case for significant action to address inequalities in both physical and mental health. Below, we offer further points of support, critique and clarification in the spirit of co-operation, which appears to be central to the agenda of Rose and colleagues.

 The open letter is particularly useful in drawing renewed attention to how necessary it is to remind decision-makers of the need for structural changes to ensure equity in health for all. Many of the key arguments posed in the letter have also been evident in emergency management and community psychology literatures for some time. These literatures document how adverse socio-economic conditions often worsen outcomes from natural and person-centered disasters, particularly for people who already endure vulnerabilities (Luna, 2009; Tierney, 2019; Wisner et al., 2004; Blake et al., 2017) ^1^^-^^4^. As is the case with other disasters, socio-political situatedness in terms of poverty, insecure work, discrimination and marginalization, and poor housing are influencing peoples’ psychological, physical, cultural and spiritual reactions to COVID-19. Therefore, it is vital to contest the unjust, social structures that perpetuate and exaggerate such adverse conditions, and champion for fairer and just solutions. We can do so by drawing on existing resources, such as the Sendai framework that offer responses to inequalities in outcomes following disasters. Such frameworks offer pathways of action to address urgent and critical responses to protect livelihoods, health and ecosystems for everyone (United Nations, 2015) ^5^.

 We agree that many of the well-publicized predictions of a ‘tsunami’ of mental health concerns stemming from the present pandemic may well be overstated. Rose and colleagues rightfully assert that, regardless, it is important to address issues around resourcing and community development, and to stretch our collective efforts towards socio-economic transformations. It is also necessary to note that, due to ongoing inequities and a lack of adequate response to the consequences of everyday injustices (poverty and discrimination), mental illness is already endemic in many countries. Systems of response also need to be re-designed to address the inverse care law (Hart, 1971) ^6^ whereby people who need care the least consume the most. Conversely, people who need care the most, access the least.

 Increases in service access proposed by Rose and colleagues are urgent. It is important to put initiatives in place to help people in distress to cope by enhancing early intervention and peer-support systems. Training more mental health professionals to meet the existing complex needs of diverse communities is crucially important. Our concern with an emphasis on therapeutic training and interventions that predominantly target individuals in distress is not to question its effectiveness in addressing certain difficulties such as trauma, anxiety and low mood. Nor is the only challenge the limited reach (i.e., the number of people that can be assisted) of this strategy and issues of scalability. Our concern relates to how such responses are predominately ameliorative, rather than preventative in orientation. Responses to wide-scale human suffering that *only* focus on therapeutic interventions should be treated with suspicion as often conservative, bureaucratic, and as a failure of political imagination. The fixation on therapeutic responses can act as an ‘action distraction’, which contributes to a fixation on responses to reduce suffering within individuals. This can result in the pacification and obscuring of calls for wider reaching efforts to promote community level wellness, and to prevent mental ill-health through preventive macro structural changes. To address health inequities and differentials across persons and population groups, we must also address the structural inequities that drive ill-health. We need to look further ‘upstream’ and prevent people from becoming ill (Hodgetts & Stolte, 2017) ^7^. Increased access to therapeutic interventions needs to be positioned overtly as part of larger strategies towards systemic change. We applaud Rose and colleagues for also raising the need for a broader change agenda.

 As Rose and colleagues also propose, redistributive policies and practices are important in addressing issues of inequity and health inequalities. In doing so, these authors adopt a well-worn population health position that is enshrined in many nation states within graduated taxation and cash transfer systems. We would add that, the levels of inequity that many communities now face, despite the existence of such redistributive initiatives, suggests the need to consider other more radical strategies for change. After all, it is in historical moments like the present that it becomes more possible to seize the initiative and to consider rebooting the whole socio-economic system (neoliberalism), which is driving inequities in life chances and health. In this context, increases in redistributive policies can be re-imagined as ‘stopgap measures’ that are useful whilst we formulate more sustainable and transformative systems for ensuring equity, social justice and health for all. This requires us to ask if, for example, now is the time for jubilee? Is now the time to introduce policies such as universal basic incomes, maximum wage settings, and resource co-ownership models? Should the ancient concept of the commons (shared resources that are available to humanity) be embraced as a central principle for a more just economic system (Standing, 2019) ^8^? What we are signaling here is the need to be even bolder and to broaden the conversation regarding how to relieve people from debt servitude and ensure nobody has to subject themselves to often abusive and dehumanizing penal welfare and social service systems (Hodgetts & Stolte, 2017) ^7^. Such structural transformations may actually reduce the need for therapists and such expensive service systems. We should be asking more questions to which we do not have perfect answers.

 One avenue for addressing discrimination in the health, penal welfare and social services nexus in many nation states is to disassemble these systems and to provide the resources they consume directly to people in need. If people are resourced to govern their own affairs collectively, the chances of their flourishing alongside others are increased. This line of reasoning speaks to issues of self-determination and is a feature of calls from indigenous groups, in particular, to (re)gain guardianship over the resources that have been taken from them. Additionally, in addressing issues of inequality and discrimination in present systems, it is important to not limit our historical gaze. For example, Rose and colleagues assert that economic supports have been stripped away over the last decade. We agree, and would also foreground the impacts of longer time scales of dispossession and inequality that have set the stage for injustices in many nation states today. The present crises in many countries have been shaped by Neoliberalism over the past 40 years. These crises are also the products of historical processes of colonization/imperialism that continue to negatively impact minoritized communities.

 Relatedly, responses called for by Rose and colleagues will need to be reformulated in context specific ways. What is appropriate for the United Kingdom in addressing issues of diversity may not necessarily be appropriate in contexts such as Aotearoa New Zealand. In our context, the English version of the Treaty of Waitangi [1] (signed in 1840 by the British Crown and Māori leaders) set the stage for the dispossession of land, resources, power and cultural practices from Māori. Subsequent exploitation by the Crown has resulted in ongoing structural inequities. As such, systemic changes will need to include the enactment of genuine Treaty of Waitangi principles that are not exploitative and extractive. This could mean that, for example, iwi (tribes) exercise guardianship over the provision of health, social and education services for their members, and for other people residing within their tribal boundaries. This would likely result in a refocusing of initiatives away from short-term economic returns and towards a longer-term, inclusive and equitable strategic focus. For instance, Māori often operate from the position of intergenerational planning, capacities and relationships whereby certain decision-making roles are assigned to kaumatua (elders) as wisdom holders, alongside often middle aged "executive leaders" (enactors) who are supported by rangatahi (youth), and inspired by pēpi (infants). More broadly, such transformations are about honouring localised treaties and embracing indigenous rights and knowledges through enactments of generational change strategies designed to achieve healthy futures for all.

 We are surprised that the open letter appears to take on a somewhat executive tone and does not feature the language of collaboration as much as we feel is warranted. From our perspective, achieving effective change requires us to look out beyond our own expertise, models and journals. Recognising the socio-political situatedness of people’s lives and who gets to have a voice in decision-making processes is particularly crucial during and after disasters. Rasche (2018) ^9^ reminds us that the social location and expertise of speakers often brings about possibilities to become an authority on other peoples’ situations. It is important that we engage directly with people who are adversely affected by the structures and issues to which we are trying to find effect responses. Policies and systems that are co-designed with the people being targeted tend to be more effective. To work effectively with others, it is also important we decentre our own ‘expert’ voices in order to hear the voices of others who have gained phronetic wisdom from having to carry the weight of adversity. Part of our shared change agenda must be to address inequities regarding who gets to be central to conversations and initiatives for change, and whose knowledge is valued and heard in these processes. We cannot leave the formulation and implementation of change strategies to the usual suspects.

 We are sure Rose and colleagues would agree that there are considerable sensitivities in considering efforts to work in partnership across dominant and marginalized communities. In the process, it is important to avoid falling into the trap of using labels that collapse diverse communities together as the ‘others’ (Fakim & Macaulay, 2020) ^10^. For example, the BAME acronym used by Rose and colleagues is considered highly problematic by many members of the diverse groups that are supposedly ‘encapsulated’ in this term. For many, BAME is a distancing, cold and administrative ‘white term’. It homogenises and simplifies the heterogeneity of the intertwined histories of adversity that are experienced in varying ways by diverse groups of people. Such terms also act as apolitical euphemisms for historically politicised and reclaimed notions, such as Black, queer and indigenous.

 A key message in our response is that what we need to advocate collectively is not only more equitable access to mental health and community services, but also access to justice, sustainable incomes, adequate housing, good food, and supportive and violence-free relationships. As Rose and colleagues note, we need to ‘build back better’. This requires us to unpack whose notion of ‘better’ is being advocated. Who gets to decide what we mean by the creation of societies that are more equitable and offer fairer access to resources, so as those necessary to promote and sustain human flourishing. We were surprised that in asserting the need to build back better, Rose and colleagues did not refer to existing global structures that offer avenues for negotiating and enacting shared visions for humanity and wellness.

 There is an opportunity to link our efforts in with the 17 interwoven United Nations Sustainable Development Goals (SDGs). Widely consulted and signed-up to by stakeholder groups from almost 200 United Nations, the SDGs have been in effect since 2016 and will run till 2030. Directly relevant to our present dialogue, psychologists specifically fought to have mental health included in this global development agenda, under SDG-3. Of the remaining 16 SDGs, particularly important are SDG-17 (Partnerships for development whereby those targeted are positioned centrally in the formulation, design and implementation of change processes) and SDG-8 (Decent Work for all, which includes access to Sustainable Livelihoods and Wellbeing at Work). In advocating for change it is important to avoid re-inventing the wheel. As imperfect as they are, the 17 SDGs offer a platform for change on a global scale and in ways that preserve our geographical specificities. The wider point of these goals, and other structures like the Global Compact and the Sendai Framework, is that they offer accountability structures that can help determine the distribution of resources and opportunities for action within and across nation states. These initiatives can be harnessed to help us redress structural inequities, injustices, exclusions and violations of Human Rights.

 We would like to thank Wellcome Open Research for providing this opportunity to respond to the seminal open letter by Rose and colleagues.

 [1] There are English and Māori or Te Reo versions of the treaty and complexities around translation.

Does the article adequately reference differing views and opinions?

Yes

Are all factual statements correct, and are statements and arguments made adequately supported by citations?

Yes

Is the Open Letter written in accessible language?

Yes

Where applicable, are recommendations and next steps explained clearly for others to follow?

Yes

Is the rationale for the Open Letter provided in sufficient detail?

Yes

Reviewer Expertise:

NA

We confirm that we have read this submission and believe that we have an appropriate level of expertise to confirm that it is of an acceptable scientific standard.

## References

[1] : Elucidating the concept of vulnerability: Layers not labels. *IJFAB: International Journal of Feminist Approaches to Bioethics*.2009;2(1) : 10.3138/ijfab.2.1.121 121-139 10.3138/ijfab.2.1.121

[2] : Disasters: A sociological approach. *Polity Books*.2019;

[3] : At risk: Natural hazards, people's vulnerability and disasters. *Routledge*.2004;

[4] : Get prepared: Discourse for the privileged?. *International Journal of Disaster Risk Reduction*.2017;25: 10.1016/j.ijdrr.2017.09.012 283-288 10.1016/j.ijdrr.2017.09.012

[5] : Sendai Framework for Disaster Risk Reduction 2015-2030.2015; Reference source

[6] : THE INVERSE CARE LAW. *The Lancet*.1971;297(7696) : 10.1016/S0140-6736(71)92410-X 405-412 10.1016/S0140-6736(71)92410-X 4100731

[7] : Urban poverty and health inequalities: A relational approach. *Routledge*.2017;

[8] : Plunder of the Commons: A Manifesto for Sharing Public Wealth. *Pelican*.2019;

[9] : “Speaking on Behalf of…”: Leadership Ethics and the Collective Nature of Moral Reflection. *Journal of Business Ethics*.2020;163(1) : 10.1007/s10551-019-04325-2 13-22 10.1007/s10551-019-04325-2

[10] : 'Don't call me BAME': Why some people are rejecting the term.June 2020; Reference source

# Peer review: Reviewer response for version 1

**DOI:** 10.21956/wellcomeopenres.17697.r39765

**Published:** 2020-08-06

**Authors:** Kim Hopper

**Affiliations:** 1 Department of Sociomedical Sciences, Mailman School of Public Health, Columbia University, New York, NY, USA

This is a fiercely argued brief for structurally informed corrective action during the pandemic, issued by an impressive group of petitioners. In eight evidence-sourced paragraphs, it manages to deliver both an indictment – the predictably unequal impact of COVID-19 on already disadvantaged groups – and an urgent call to civic arms – directed by a hybrid of technical expertise (from social and clinical sciences) and vernacular knowledge. It returns concerns about mental health effects to more general considerations of everyday securities and de-stigmatizes the relief and prevention efforts called for. In sum and substance, it converts an analysis of epidemiological contours into the warrant for social reform such an analysis has (at least since the time of Virchow) regularly invited but rarely entrained. The “rebuilding” agenda itemized here has an urgency and scope that are radical and entirely reasonable; it owns, not refuses, the subtext that haunts the daily bills of mortality.

Does the article adequately reference differing views and opinions?

Yes

Are all factual statements correct, and are statements and arguments made adequately supported by citations?

Yes

Is the Open Letter written in accessible language?

Yes

Where applicable, are recommendations and next steps explained clearly for others to follow?

Yes

Is the rationale for the Open Letter provided in sufficient detail?

Yes

Reviewer Expertise:

Public and cross-cultural mental health; social determinants of population health.

I confirm that I have read this submission and believe that I have an appropriate level of expertise to confirm that it is of an acceptable scientific standard.

# Peer review: Reviewer response for version 1

**DOI:** 10.21956/wellcomeopenres.17697.r39549

**Published:** 2020-07-15

**Authors:** Peter Yellowlees

**Affiliations:** 1 Department of Psychiatry, UC Davis School of Medicine, Sacramento, CA, USA

This is a well written and presented opinion piece from a large group of multidisciplinary experts. The authors argue for the need to focus on refunding and resupporting social and mental health systems which have been defunded in recent years, as a more appropriate response to the mental health needs identified by the current pandemic, rather than focusing primarily on providing increased acute services. They make the important point that the pandemic will most severely affect those who are already most socially excluded, namely the Black, Asian and minority ethnic communities, and describe a series of useful "building blocks" for building mental health back better and fairer in the post pandemic world.

Does the article adequately reference differing views and opinions?

Yes

Are all factual statements correct, and are statements and arguments made adequately supported by citations?

Yes

Is the Open Letter written in accessible language?

Yes

Where applicable, are recommendations and next steps explained clearly for others to follow?

Yes

Is the rationale for the Open Letter provided in sufficient detail?

Yes

Reviewer Expertise:

Mental health services research.

I confirm that I have read this submission and believe that I have an appropriate level of expertise to confirm that it is of an acceptable scientific standard.
